# Dopaminergic computations for perceptual decisions

**DOI:** 10.1016/j.cobeha.2024.101458

**Published:** 2024-12

**Authors:** Samuel Liebana, Matthias Fritsche, Armin Lak

**Affiliations:** Department of Physiology, Anatomy & Genetics, University of Oxford, Oxford, United Kingdom

**Keywords:** Perception, Reward, Learning, Dopamine, Reinforcement Learning, Confidence, Model

## Abstract

Studies linking the brain’s dopamine signals with learning and decision making have enjoyed enormous progress using predominantly value-based decision-making tasks. However, recent studies have demonstrated pervasive dopamine signaling also during perceptual decision making. These signals have been shown to depend on both feedback and perceptual parameters such as perceptual decision confidence and sensory statistics. Here, we review recent studies investigating dopamine signals in simple and complex forms of perceptual decision tasks across species and dopaminergic circuits. We discuss how reinforcement learning (RL) models can account for key aspects of learning during perceptual decision making and its dopaminergic underpinnings, thus bridging the gap with the literature on dopamine in value-based decisions. Finally, we propose that RL may provide a promising framework to address current challenges in the dopamine literature, such as explaining the function of its heterogeneous responses and its role in learning from naive to expert.

## Introduction

**Figure 1:**
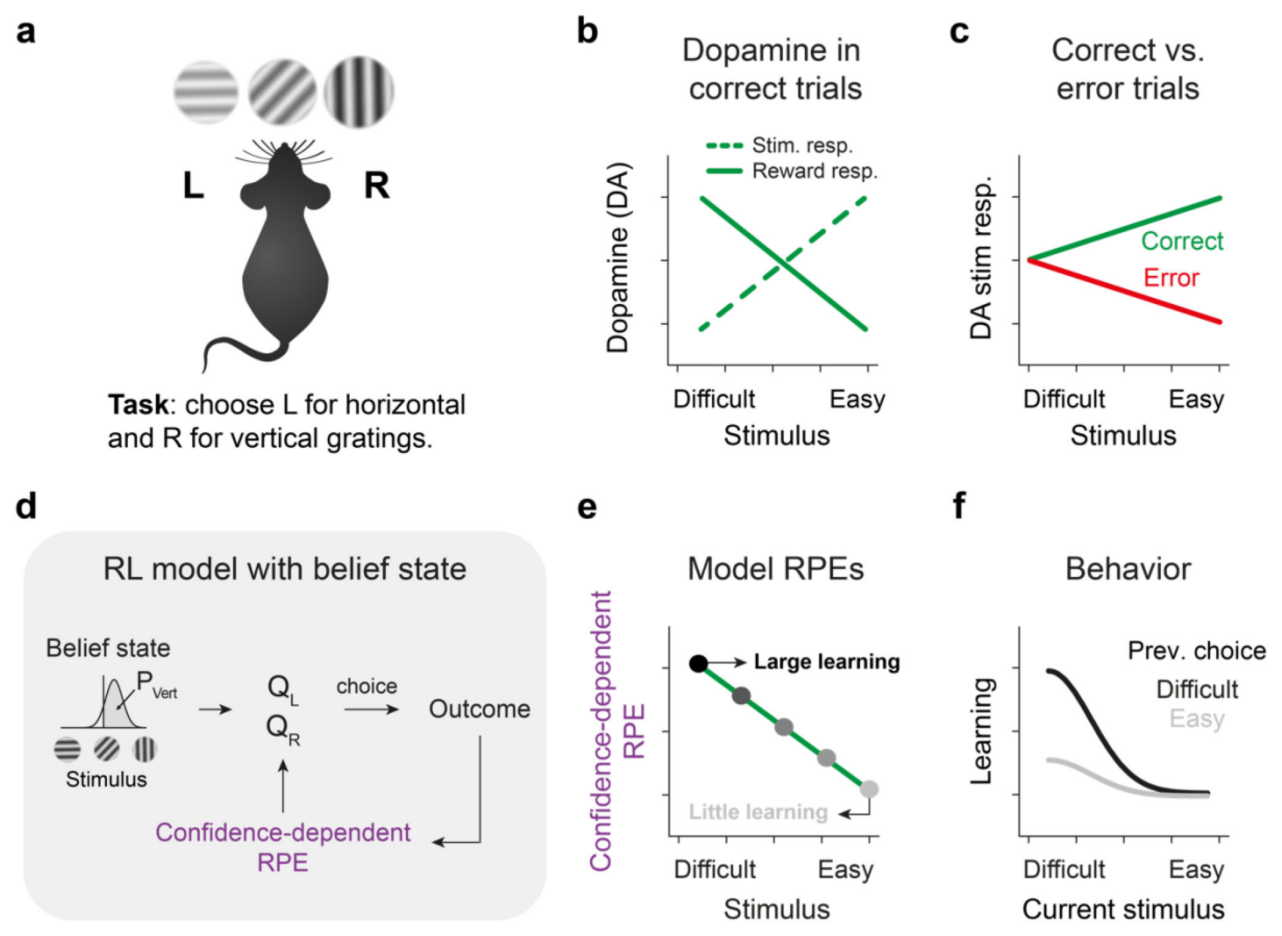
Dopamine signals during perceptual decision making can be explained with RL. (a) Schematic of a perceptual decision-making task. (b) Relation between perceptual evidence and dopamine responses to stimulus and reward on correct trials. (c) Relation between perceptual evidence and stimulus-evoked dopamine on correct and error trials. (d) RL model learning action values Q_L_ and Q_R_ through confidence-dependent RPEs incorporating belief state estimates. (e) Relation between perceptual evidence and confidence-dependent RPEs. (f) Effect of confidence-dependent RPEs on behavior.

Every time we look at a traffic light to decide whether to cross the street, listen to the washing machine to know whether it has finished, or smell the oven to check if our cake has burnt, we are making a perceptual decision. Perceptual decision making refers to the process of detecting, discriminating or categorizing often noisy (uncertain) sensory information to guide behavior (Fig 1a). Such perceptual decisions have been classically conceptualized within a statistical, psychometric framework [1,2] where the properties of sensory signals are the principal determinant of performance. This framework has also been instrumental in understanding how neural responses of sensory systems evolve as individuals learn and refine their perceptual decisions [3–5]. However, a crucial part of perceptual learning relies on whether past decisions were successful, highlighting reward as an important factor in refining perceptual decisions [6–11]. Even more pronouncedly, naive animals learning to make perceptual decisions are motivated primarily by rewards. Recent studies have started to investigate the effects of such reward-based learning on perceptual decisions, revealing interactions which explain multiple behavioral and neural phenomena. In this paper, we review these studies with a particular focus on dopamine signals underlying learning during perceptual decision making and computational models that account for related empirical observations.

Perceptual and reward-based learning have traditionally been presented as two distinct processes engaging separate brain mechanisms. The former is mainly associated with sensory and association cortical circuits involved in perception and evidence integration [4], whereas the latter is concerned with frontal cortex and subcortical regions such as striatum and midbrain dopamine neurons [12–14]. Owing to this separation, most studies investigating dopamine signals have been performed in so-called value-based (i.e. reward history-guided) decision tasks, where either no sensory stimuli are presented and animals choose actions based on reward history (e.g. two-arm bandit tasks) or the stimuli are readily distinguishable and animals track their ‘value’ based on association with past rewards [15,14]. The reinforcement learning (RL) framework has been immensely successful at explaining both behavioral and neural results from these experiments [16–18]. Phasic dopamine responses closely resemble the reward prediction error (RPE) term of RL models - the moment-by-moment difference between predicted and realized reward - which can be used as a teaching signal for learning to improve subsequent decisions [19–25].

Notably however, dopamine neurons are also active during perceptual decisions. In perceptual decision-making experiments, reward history alone does not provide an accurate estimate of upcoming outcome value. Instead, accurate decisions (and predictions about their outcomes) additionally require the evaluation of available sensory evidence, e.g., discriminating between different sensory cues or detecting the presence/absence of a cue, which can be difficult due to weak sensory signals and perceptual noise (Fig 1a). Further, stimuli can be parametrically manipulated to enable precise control over sensory variables and investigate their effect on decision making. Studies across species and sensory modalities have shown that dopamine neurons, alongside several other nodes across the basal ganglia, respond to sensory stimuli and decision outcomes during perceptual decision making [26–30]. Generally, it has been observed that dopamine signals increase with the level of sensory evidence during stimulus processing, and conversely decrease with sensory evidence during outcomes (Fig 1b). For instance, in a visual random dot motion discrimination task, the spiking activity of midbrain dopamine neurons of non-human primates increased with motion coherence during stimulus presentation and decreased with motion coherence during reward delivery [26,31]. Likewise, in non-human primates performing a somatosensory go/no-go task, the spiking activity of midbrain dopamine neurons increased with stimulus vibration amplitude [27]. Dopamine-specific imaging of midbrain dopamine neurons in mice performing a visual contrast detection task revealed responses that were scaled by the contrast of the stimuli, being largest for high contrast stimuli - a relationship that once again reversed during reward delivery [28]. Finally, monitoring dopamine release in different striatal regions in mice has confirmed a positive scaling of dopamine release with the strength of stimuli in visual, olfactory and auditory decision tasks [32–36]. Across many of these studies, the dopamine responses to stimuli encoded the accuracy of the pending choice: they were lower in error trials compared to correct trials after controlling for stimulus strength (Fig 1c). These observations suggest that dopamine encodes the subjective sensory experience and perceptual decision uncertainty rather than the physical strength of sensory stimuli. Together, there is abundant evidence that dopamine signals are modulated by the strength of perceptual evidence during stimulus and feedback processing.

The ubiquitous nature of dopamine responses during perceptual decision making begs the question of what role they play in this process. Are they important for the current decision process or subsequent learning? Dopamine responses to stimuli are present prior to the behavioral manifestation of a perceptual decision, e.g. a saccade [31]. However, consistent with value-based studies [37], these signals do not appear to have substantial and consistent effects on impending decisions. Optogenetic manipulation of dopamine signals during stimulus processing has provided differing results: manipulating midbrain dopamine neurons did not influence impending visual decisions [28], but manipulating dopamine axons projecting to posterior striatum influenced auditory decisions [38]. On the other hand, recent studies in animals refining their decisions have suggested that dopamine signals play a significant role in *learning* to make perceptual decisions [28]. In fact, framing perceptual decision making as a reinforcement learning problem makes testable predictions for how perceptual decisions depend on past decisions and dopamine signals, and how dopamine signals are modulated by perceptual parameters, which we review below.

### Perceptual decision making as a reinforcement learning problem

Under reinforcement learning (RL) formalism, the challenge of an agent faced with a perceptual decision is to infer the state of the world from the incoming sensory signals in order to select an appropriate choice. Given the ambiguity in the sensory information, such state inference is inherently probabilistic, requiring the calculation of a *belief state* (Fig 1d), as conceptualized in RL models using partially observable Markov decision processes (POMDP) [39]. A belief state is a subjective trial-by-trial estimate of the probability that any particular state is the true state, based on which a statistical decision confidence can be estimated. This statistical decision confidence reflects the probability that the pending choice will be correct given the sensory evidence. RL models incorporating such belief states yield reward prediction errors scaled by decision confidence, i.e. confidence-dependent RPEs (Fig 1d). This state inference problem, and the resultant confidence-dependent RPEs, are present even in well-trained individuals as long as the sensory evidence is ambiguous.

If such confidence-dependent RPEs guide the trial-by-trial learning of perceptual decisions, they should have a specific and testable effect on trial-by-trial fluctuations in perceptual decisions. In particular, these fluctuations should depend on decision confidence: following a rewarded difficult decision based on ambiguous sensory evidence, the RPE is substantially larger than after rewarded easy decisions (Fig 1e). This larger RPE signal should update action values to create a larger bias (side shift) in the psychometric curve towards the past rewarded choice. Furthermore, this shift should be most prominent when the current sensory evidence is weak (Fig 1f). Recent studies have shown that this prediction indeed holds in different perceptual decision tasks across sensory modalities and across species [10,40]. Such confidence-dependent learning is not due to low-level sensory adaptation [36]. Consistently, psychometric shifts are present regardless of the modality of the preceding perceptual decision (e.g. in a task in which auditory and olfactory stimuli are randomly interleaved), as long as the decision was difficult [10]. RL models can account for this by storing and updating the value of actions (e.g. left and right) independently of the modality of the stimuli. This confidence-dependent learning ensures that more learning occurs in the most informative trials, i.e. when the individual makes a correct decision in response to a difficult stimulus.

### Dopamine signals and confidence-dependent learning

Confidence-dependent RPEs account for several facets of dopamine signaling during perceptual decisions. In particular, the positive scaling of dopamine with sensory evidence during stimulus processing is consistent with an increased prediction of reward when perceptual confidence is high. Conversely, the reverse scaling during reward processing - low dopamine for rewards following decisions based on certain sensory evidence - is consistent with reduced reward prediction errors after correct high-confidence decisions (Fig 1b c.f. Fig 1e). Supporting the idea that these dopaminergic RPEs drive learning, it has been found that manipulating post-outcome dopamine responses indeed induces psychometric shifts in subsequent perceptual decisions consistent with the RL model [28]. Together, these results suggest that dopamine signals underlie the trial-by-trial learning observed during perceptual decisions.

In natural contexts, perceptual decisions not only depend on current sensory information, but also on recent perceptual observations. Indeed, our natural environment exhibits a multitude of temporal regularities among stimuli which can be exploited to predict the future from the recent past, thereby facilitating perceptual decisions. Past studies have demonstrated that observers adapt their perceptual choice history biases to such temporal regularities. For example, humans and animals tend to adaptively repeat or alternate choices in environments in which sensory stimuli are likely to repeat or alternate [6,41–43]. Recent experiments have found that striatal dopamine may reflect the temporal regularities of stimuli and track the adaptive perceptual choice history biases [36]. In particular, dopamine release in the dorsolateral striatum tracks the RPEs of a reinforcement learning model which, in addition to confidence-dependent learning of associations between current stimuli and actions, also retains and updates the relationship between a memory of past stimuli and current actions. This suggests that striatal dopamine might not only be involved in associating actions with current sensory stimuli, but also with recent perceptual history, according to the temporal regularities of the environment.

### Reinforcement learning models and current challenges in understanding dopamine

Despite the overall consistency of how dopamine signals relate to perceptual uncertainty, there is considerable heterogeneity in the tuning of individual dopamine neurons during perceptual decision making. For instance, midbrain dopamine neurons of mice have been shown to encode a variety of sensory, motor and cognitive variables during a visual evidence integration task [44]. During a visual random dot motion task in non-human primates, some dopamine neurons encoded perceptual decision uncertainty, while others encoded the expected magnitude of reward associated with perceptual decisions [31]. Dopamine functional heterogeneity also appears to depend on dopaminergic axonal projection pathways. For instance, in a visual contrast detection task for mice, dopamine axons in the dorsomedial striatum only responded to contralateral, but not ipsilateral visual stimuli [33], similar to the contralateral choice tuning observed in value-based decision-making tasks [45]. Instead, dopamine axons in the ventral striatum showed contrast-dependent responses to both contralateral and ipsilateral stimuli [33]. This projection-specific heterogeneity may vary throughout the perceptual decision-making process. For example, a study examining dopamine release in the ventral and posterior (tail) striatum during an auditory decision task found a similar representation of sensory evidence in both striatal regions, but projection-specific encoding of reward and stimulus expectations in pre-stimulus epochs [34]. Lastly, compared to the relative consistency of studies examining dopamine signals in dorsal or ventral striatum, studies investigating dopamine signals in the tail of the striatum have argued for differing roles of these signals, including stimulus expectation, action prediction error and stimulus novelty [46–48]. Although the heterogeneity of dopamine responses is often presented as an argument against RPE encoding, recent studies have proposed anatomically plausible models which suggest that it may be compatible with the RL framework [25,49,50]. These models explain dopaminergic heterogeneity with the concept of ‘partial’ or ‘feature-specific’ RPEs, which reflect the difference between a reward prediction calculated using a subset of environmental features and a global reward signal. These augmentations to the RL framework can be used to understand the functional relevance of observations such as dopaminergic heterogeneity, and may also inform the design of artificial agents in machine learning.

Learning to make perceptual decisions from naive to expert is a complex problem often requiring long periods of time. Our understanding of the role of dopamine in such long-term learning has seen little progress in the past, as most studies have focused on well-trained animals refining their decisions over short timescales. Long-term learning presents several challenges for an individual, and understanding how the brain tackles these could yield important insights into the brain's learning mechanisms. For example, one of the first learning problems for a naive individual is to discover which stimuli (states) are relevant for the task. Recent studies provide evidence for the progressive discovery of states in mice learning a perceptual task from naive to expert [49]. Striatal dopamine signals reflect the evolving state representations, showing key characteristics of a teaching signal sculpting individuals' learning trajectories [49,35,51]. Standard RL models commonly used to explain animal behavior cannot account for this because they assume that the relevant states have already been discovered, such that reward-driven updating of state-action associations can take place from the outset. However, a *deep* RL model could capture both the learning trajectories and dopamine signals of mice learning from naive to expert [49]. This model incorporates flexible state representations to capture the progressive state discovery and proposes that striatal dopamine reinforces associations between states and actions only if the states have been discovered. Taken together, examining dopamine signals can hence provide valuable insights into both the behavioral and neuronal bases of learning from naive to expert.

### Concluding remarks

Dopamine signals play an important role in shaping perceptual decisions and are themselves influenced by perceptual parameters such as sensory uncertainty, sensory statistics and the discovery of relevant sensory stimuli. By integrating such perceptual parameters into the computation of value, RL provides a useful framework to understand dopaminergic signals and their function during perceptual decisions, thus bridging the gap with the rich literature of dopamine in value-based decision making. Perceptual decision making also serves as an effective tool to probe dopamine signals, having revealed phenomena such as the encoding of perceptual decision confidence and dopaminergic heterogeneity. These observations have in turn informed extensions of the RL framework, enabling it to better capture animal behavior and its neural implementation. Altogether, studying dopaminergic signals in perceptual decision tasks, and viewing them through the lens of RL, has led to a better understanding of how reward-based learning shapes perceptual decisions. Excitingly, the expanding repertoire of tools to record dopamine and more complex perceptual decision-making tasks promise to deliver continual insights in this domain.
